# Reconstruction of Tokamak Plasma Emissivity Distribution by Approximation with Basis Functions

**DOI:** 10.3390/s25103162

**Published:** 2025-05-17

**Authors:** Tomasz Czarski, Maryna Chernyshova, Katarzyna Mikszuta-Michalik, Karol Malinowski

**Affiliations:** 1 National Centre for Nuclear Research, Andrzeja Sołtana 7, 05-400 Otwock, Poland; maryna.chernyshova@ncbj.gov.pl; 2 Institute of Plasma Physics and Laser Microfusion, Hery 23, 01-497 Warsaw, Poland; katarzyna.mikszuta@ifpilm.pl (K.M.-M.);

**Keywords:** plasma diagnostics, X-ray tomography, basis functions approach

## Abstract

The present study focuses on the development of a diagnostic system for measuring radiated power and core soft X-ray intensity emissions with the goal of detecting a broad spectrum of photon energies emitted from the central plasma region of the DEMO tokamak. The principal objective of the diagnostic apparatus is to deliver a comprehensive characterization of the radiation emitted by the plasma, with a particular focus on estimating the radiated power from the core region. This measurement is essential for determining and monitoring the power crossing the separatrix, which is a critical parameter controlling overall plasma performance. Since diagnostics rely on line-integrated measurements, the application of tomographic reconstruction techniques is necessary to extract spatially resolved information on core plasma radiation. This contribution presents the development of numerical algorithms addressing the problem of radiation tomography reconstruction. A robust and computationally efficient method is proposed for reconstructing the spatial distribution of plasma radiated power, with a view toward enabling real-time applications. The reconstruction methodology is based on a linear model formulated using a set of predefined basis functions, which define the radiation distribution within a specified plasma cross-section. In the initial stages of emissivity reconstruction in tokamak plasmas, it is typically assumed that the radiation distribution is dependent on magnetic flux surfaces. As a baseline approach, the plasma radiative properties are considered invariant along these surfaces and can thus be represented as one-dimensional profiles parameterized by the poloidal magnetic flux. Within this framework, the reconstruction method employs an approximation model utilizing three sets of basis functions: (i) polynomial splines, as well as Gaussian functions with (ii) sigma parameters and (iii) position parameters. The performance of the proposed method was evaluated using two synthetic radiated power emission phantoms, developed for the DEMO plasma scenario. The results indicate that the method is effective under the specified conditions.

## 1. Introduction

Effective plasma control is imperative for the successful operation of the DEMO reactor. The considerable dimensions and extreme conditions of the burning fusion plasma in the DEMO reactor present substantial challenges in terms of maintaining stable, high-performance operation over extended periods. The crucial issue facing the future tokamak reactor is the development of highly efficient thermal insulation of the plasma. This is characterized by the energy confinement time, which is determined by the balance between losses and heating power. Maintaining an appropriate balance between incident and lost power is essential for effective thermonuclear plasma control. Energy losses in tokamaks occur through multiple mechanisms, e.g., magnetohydrodynamic (MHD) modes, associated diffusion processes, non-local transport, and radiation cooling. Careful control of heavy impurity concentrations is particularly critical, as their uncontrolled accumulation can cause increased radiation losses and plasma cooling. The radiation emitted from impurities such as carbon or tungsten represents a significant pathway for power loss from fusion plasmas [1,2], making radiation distribution measurement essential.

It is well documented that X-ray diagnostics provide vital insights into a multitude of phenomena occurring within the tokamak plasma. These include MHD fluctuations and precursors to destructive instabilities due to the transport of high Z impurities interacting with MHD activity. These interactions are a fully three-dimensional phenomenon, as evidenced by, for instance, three-dimensional tungsten redistribution in NTMs or internal kink modes. They can be a consequence of, for example, plasma rotation or off-axis heating of ICRH. Extreme ultraviolet radiation (XUV) and soft X-rays (SXR) are produced by a number of processes which involve electrons interacting with ions or an external magnetic field. These processes include bremsstrahlung, cyclotron emission, radiative recombination, and the deexcitation of atoms or ions. The outgoing XUV/SXR radiation is a complex function of electron temperature, electron density, and the concentration/distribution of impurities in the plasma. Passive spectroscopy represents the most straightforward approach for the detection and investigation of the temporal evolution of magnetic confinement fusion plasma impurities. In the event that the magnetic flux surfaces can be identified as iso-emissive surfaces, then SXR can also provide information regarding the hydro magnetic structure of the plasma [3].

The presence of impurities in magnetic fusion plasmas gives rise to XUV/SXR emissions, which serve as a valuable source of information regarding the underlying physical processes occurring within the plasma. These emissions have their origin in the background plasma, which is typically composed of deuterium or hydrogen, and, on occasion, helium with some impurities. The latter result from the interaction of the main plasma with the vacuum chamber, limiter/divertor plates, antennas, or other structures within the device. The impurities present in the plasma thus depend on the specific elements with which the plasma comes into contact. These materials include graphite (C), stainless steel (Fe, Ni, Cr, etc.), Mo, W, and gases that have been absorbed by the material itself (O, Cl, Ne, N, etc.).

As indicated above, the intensity and spatial distribution of plasma radiation convey pivotal information regarding plasma properties. Additionally, plasma-related radiation is a three-dimensional phenomenon. This implies the utilization of a set of tomographic measurements for the investigation of plasma parameters. By employing tomographic reconstruction techniques on SXR data, the spatially resolved distribution of radiation emissions can be recovered, thereby providing a detailed two- or three-dimensional representation of the plasma radiation structure. This spatially resolved information is of paramount importance for validating theoretical models, optimizing plasma scenarios, and ultimately achieving the high confinement required for fusion energy production. To date, tomography utilizing line integrated data represents the principal analytical method for identifying the distributed physical parameters in fusion science and for evaluating local plasma emissivity information.

The fundamental principle of plasma radiation tomography involves measuring the integrated radiation signals along multiple lines of sight through a plasma volume. The mathematical inversion problem of obtaining local emissivity of the plasma from measurements taken by a set of detectors can be solved by applying reasonable conditions, constraints, and regularization to reduce the degrees of freedom [4,5,6]. In practice, the application of tailored reconstruction methods is contingent upon the imposition of constraints, which are necessary to facilitate the inversion task and for the selection of reasonable solutions. Obtaining accurate and robust tomographic reconstruction is paramount for extracting reliable plasma information from radiation measurement data. However, this inverse problem is inherently ill-posed and underdetermined, presenting significant challenges in terms of the uniqueness, stability, and resolution of the reconstructed emissivity profiles [7,8]. In order to address these challenges, a number of reconstruction techniques have been developed, each with its own specific strengths, limitations, and fundamental assumptions. Additionally, a variety of inversion techniques have also been developed for plasma tomography across different energy ranges, including VUV, SXR, hard X-rays, and gamma rays [1,2,3,7,8,9].

This paper proposes a deterministic method using basis function approximation to develop a robust, sufficiently fast technique for real-time estimation of core plasma radiation power—a necessity for plasma control in the future DEMO plant. While the general case requires an extensive detector set, we applied initial assumptions related to the magnetic field to simplify the process. The proposed method yielded promising and satisfactory results.

## 2. Motivation for the Proposed Tomographic Reconstruction Method

The described approach was chosen based upon the requirements of the plasma fusion domain with regard to real-time plasma control in future reactors, particularly in the context of the DEMO fusion power plant. As noted earlier, this study focuses on the development of a diagnostic system designed to measure radiated power and core SXR emissions in the DEMO tokamak [10,11,12,13]. The system aims to provide a comprehensive characterization of central plasma radiation, with emphasis on estimating core radiated power. The information obtained from the diagnostics will be employed for the purpose of providing feedback, which will then be used as input for the plasma actuators. Consequently, devices such as DEMO require the implementation of straightforward, rapid, yet reliable and trustworthy algorithms and methodologies for the determination of plasma parameters in real time. The aforementioned set of basis functions represents one such proposed solution. There is some literature using this method [9,14,15,16,17,18,19]. Typically, different functions and implementation methods are used depending on the circumstances. However, direct comparisons require the same conditions and circumstances for images and detection systems, which is not always possible.

**Figure 1:**
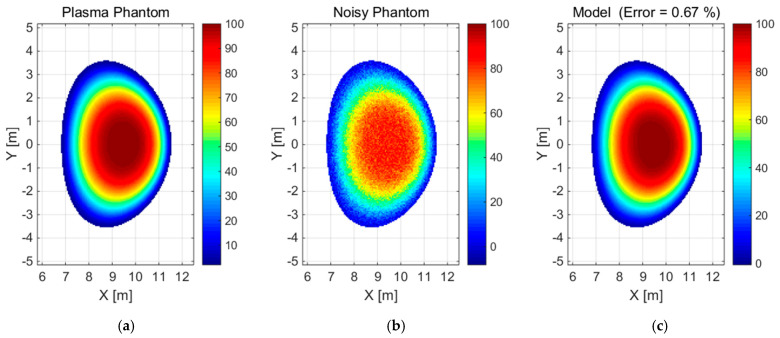
(**a**) Image of the convex phantom, (**b**) the 5% noisy phantom, and (**c**) the corresponding model of radiation distribution for the plasma radiation phantom (**a**) including additional noise (**b**).

**Figure 2:**
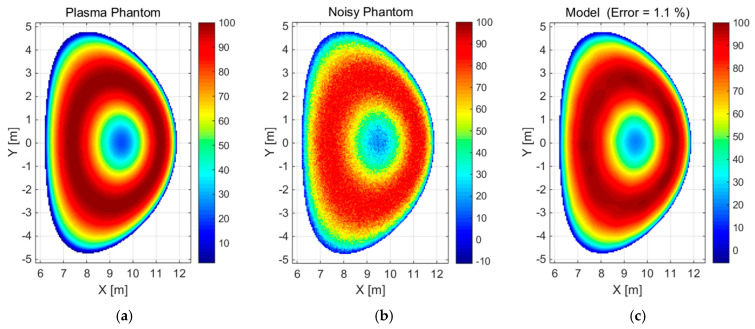
(**a**) Image of the concave phantom, (**b**) the 5% noisy phantom, and (**c**) the corresponding model of radiation distribution for the plasma radiation phantom (**a**) including additional noise (**b**).

The use of basis functions is pervasive in the approximation, reconstruction, and smoothing of noisy signals. In this study, the assumptions underlying the reconstruction method based on modelling with basis functions have been validated. From a mathematical perspective, this model can be understood as an orthogonal projection of the original onto a linear space generated by basis functions [20]. In theory, any set of linearly independent functions can serve as a basis for a linear space generated by them; however, not all are equally effective. In practice, the choice of basis functions is arbitrary, depending on the nature and specificity of the signals in question. Mathematically, the ideal basis functions are Dirac functions, to which Gaussian functions tend to converge as σ → 0. Consequently, a practical approximation is provided by uniformly distributed planar Gaussian functions, which constitute the basis for the reconstruction of the plasma emissivity distribution. The reconstruction method was evaluated through the analysis of a series of simulations of plasma images. In order to test the efficacy of the proposed methodology, two radiation power emission phantoms were subjected to analysis: a convex (Figure 1a) and a concave (Figure 2a) type, within a grid of 134 × 206 measurement points with a resolution of 5 cm (2018 EU-DEMO baseline scenario). Prior to commencing tomographic reconstructions, it was necessary to confirm the validity of the assumptions inherent to the method itself. This was accomplished by effectively approximating the aforementioned reference radiation distributions (Figure 1a and Figure 2a) using a set of Gaussian functions as basis functions. Moreover, a model based on the selected basis functions of the referenced radiation distribution was prepared, as shown in Figure 1c and Figure 2c. This followed the introduction of normally distributed noise (displayed in Figure 1b and Figure 2b) with a mean of 0 and a standard deviation of 5% of the maximum emissivity value. The noisy convex phantom was reproduced by the model based on 108 Gaussian functions with an error/relative difference of 0.67% in terms of the norm compared to the reference data (see Figure 1). The noisy concave phantom was reproduced by the model based on 1145 Gaussian functions with a relative difference of 1.1% (see Figure 2). The parameters of the Gaussian base functions, namely *position* and *sigma*, were appropriately selected in the automatic review process for a given set.

Since the reconstruction of an unknown object is fundamentally based on its model, these concepts are considered practically equivalent and are used interchangeably throughout the remainder of this article where contextually appropriate.

## 3. Theoretical Fundamentals of the Tomography Method

**Figure 3:**
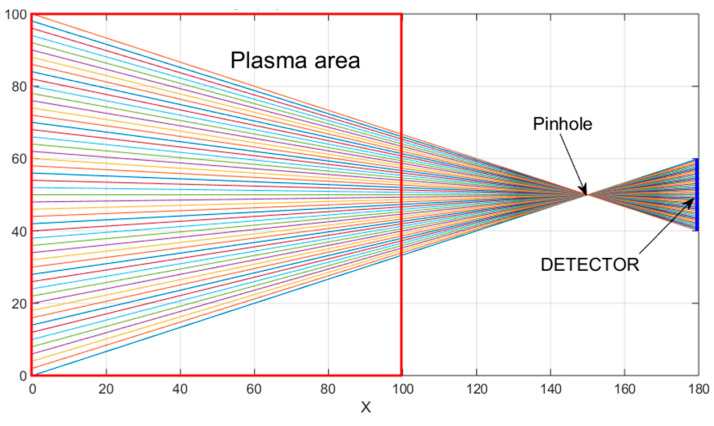
The topography for a set of N = 50 detectors, designed for the purpose of recording radiation from the plasma region in a normalized square.

Tomography is a technique used to reproduce the spatial distribution of plasma radiation power/intensity based on measurements from external detectors that record the cumulative effect from a given direction via line of sight. Figure 3 illustrates a measurement diagram for a set of detectors that record radiation within a plasma cross-section through a small pinhole [4,5,6,19,21].

The cumulative effect of radiation from a specific angular sector, $S_{n}$, is recorded by each nth segment of the detector. Consequently, for N detectors, a set of N measurement data {$Z_{n}$} is obtained, presented as a vector $Z\left[N\times 1\right]$. The planar distribution of the radiation intensity W(P) at points P of the plasma cross-section represents the primary image for the transformation through the pinhole of the detection system. Intensity distribution **Z** on the detection line is therefore a projection of the planar plasma image W, as a transformation through contribution operator **H**:

(1) $$Z=H\left(W\right)$$

In particular, the set of values $W\left(P∊S_{n}\right)$ for all points P from the nth angular sector $S_{n}$ is mapped into the measurement value for the nth detector $Z_{n}$. The proposed method for reconstructing the radiation distribution $W\left(P\right)$ at points P of a given plasma cross-section is based on linear planar distribution model $w\left(P\right)$ described by a data set of base functions ${\{E}_{k}\left(P\right)$}. The resulting intensity in the plasma cross-section is modelled by a linear combination of K base functions for a set of K coefficients {$a_{k}$}:

(2) $$W\left(P\right)≅w\left(P\right)=\sum_{k=1}^{K}a_{k}E_{k}\left(P\right).$$

A discrete model defined by vectors $W\left(P\right)\left[M\times 1\right]$ and $w\left(P\right)\left[M\times 1\right]$ for M points P of the measurement grid can be represented by a set of base functions as a matrix $E\left(P\right)\left[M\times K\right]$ for K component vectors **E**_k_(P). The set of unknown coefficients {$a_{k}$} in Equation (2) constitutes a vector $A\left[K\times 1\right]$. Finally, Equation (2) is represented in matrix form:

(3) $$W\left(P\right)≅w\left(P\right)=E\left(P\right)\cdot A.$$

This results in a significant reduction of the original number M of unknown values at points P to the number K of unknown coefficients for the given base functions.

For the linear operator (matrix) **H**$\left[N\times M\right]$, the detector image of the plasma is obtained from Equation (1) and the model image from Equation (3), while measurement vector **Z** is compared with detection model vector z:

(4) Z≅z=H·w=H·E·A=G·A.

The result of mapping $E\left[M\times K\right]\overset{H}{\Rightarrow }G\left[N\times K\right]$ is matrix G=H·E. In a system comprising N detectors for K base functions, the matrix $G\left[N\times K\right]$ represents the mapping. In particular, for the nth detector and the k-component, vector $E\left(P∊S_{n},k\right)$ is mapped to a scalar $G\left(n,k\right)$ for all points P from the nth angular sector $S_{n}$. The matrix vectors G represent secondary base functions for the linear model of the detection vector z=G·A. The objective of tomography is to estimate vector **A**, which is unknown, based on the measurement data of vector Z. The estimation of vector **A** for the minimum norm of the error vector ||z−Z||, which is represented by the scalar product $G^{T}\cdot \left(z-Z\right)=0$, satisfies the orthogonal condition [22], where the subscript ^T^ is the transpose:

**A** = (**G**^T^
**G**)^−1^
**G**^T^
**Z**. (5)

In accordance with the least squares method, this represents the optimal solution of the system of Equation (4) in practical application for the over-determined system N > K or the under-determined system N < K. The reconstruction of the plasma distribution is achieved by applying Equation (3). In general, the efficiency of this procedure can be enhanced by introducing a more complex set of detectors that provide additional information from different directions.

## 4. Verification Scheme for the Reconstruction Method

In the reconstruction of an unknown image of plasma, only a projection of its cross-section in the detector system may be available. As a result, reproducing the original image by reverse operation is not a straightforward process and requires the application of certain constraints and simplifications. One such mathematical approximation method is the utilization of a linear space model of basis functions, which is the focus of this work. The verification procedure of the presented method is based on a comparison of the phantom simulation with the reconstructed model, as defined in the following steps of the algorithm.

1. Determining the geometry of the measurement grid points in the coordinate system $P\left(x,y\right)$.
2. Defining the contribution operator **H** as the matrix representing geometry of the detection system.
3. Simulation of the intensity distribution $W\left(P\right)$.
4. Simulation of the detector measurement **Z**(n), defined in Equation (1).
5. Modeling the distribution using a set of base functions $E\left(P,k\right)$, as defined in Equations (2) and (3).
6. Modeling the measurement using a set of base functions $G\left(n,k\right)$, as defined in Equation (4).
7. Estimation of vector **A**(k) given by Equation (5).
8. Defining the intensity distribution model **w**(P), as defined in Equation (3).
9. Comparison of the simulation of the **W** distribution with the **w** approximation, and the determination of the reconstruction error.

In general, the relative error of the approximation of vector **X** by vector **x** is defined as the ratio of the norm of the error vector to the norm of the reference vector: $\frac{\left|\left|x-X\right|\right|}{\left|\left|X\right|\right|}$.

**Figure 4:**
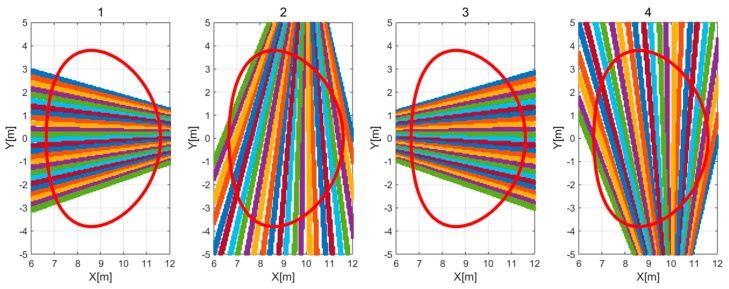
Four configurations of a system comprising 26 detectors, together with the field of view and the outline of the plasma area. The colored lines depict the lines of sight, whereas the red contour represents the last closed magnetic field surface.

**Figure 5:**
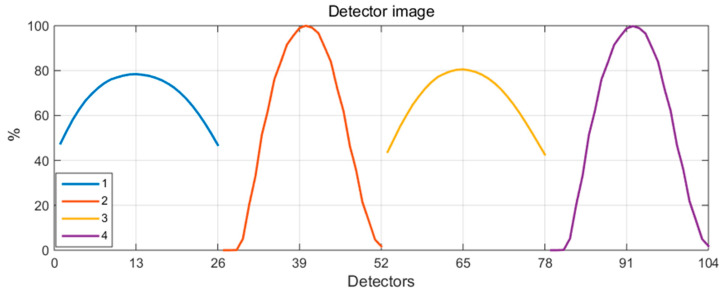
Detector images as simulated measurements for a convex phantom, corresponding to four configurations of the detection system.

**Figure 6:**
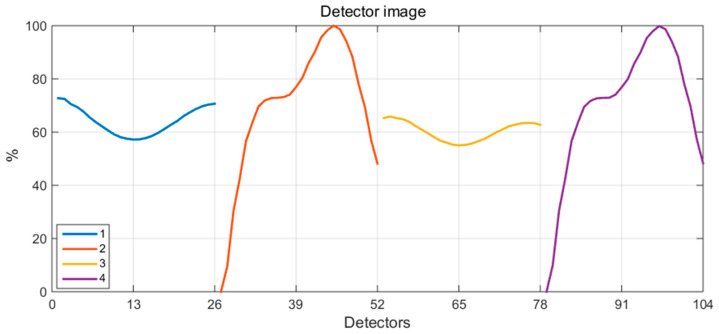
Detector images as simulated measurements for a concave phantom, corresponding to four configurations of the detection system.

Reconstruction studies were conducted on two reference plasma phantoms presented in Figure 1 and Figure 2 in a system comprising 26 detectors, arranged in various combinations of four independent configurations (illustrated in Figure 4). The resulting detector images, as simulated measurements for convex and concave phantoms corresponding to the four configurations of the detection system, are presented in Figure 5 and Figure 6, respectively, with normalized data.

**Figure 7:**
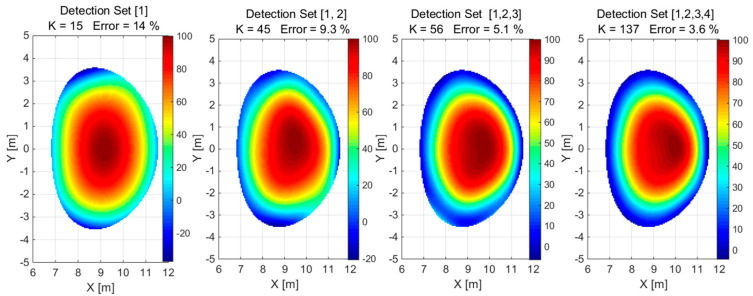
The reconstruction of the convex phantom using a detection set identified by configuration numbers in square brackets. The value of K is the number of Gaussian basis functions used for the approximation.

**Figure 8:**
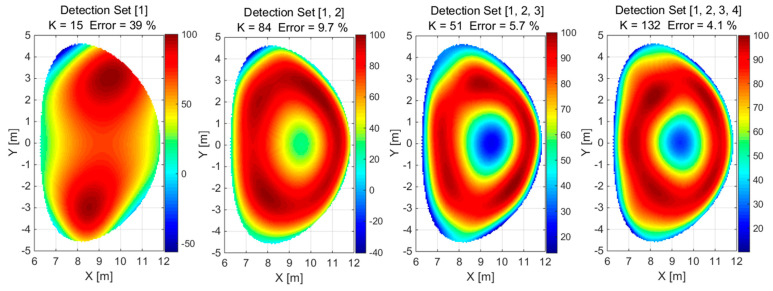
The reconstruction of the concave phantom using a detection set identified by configuration numbers in square brackets. The value of K is the number of Gaussian basis functions used for the approximation.

A combination of different detector configurations, i.e., 1 × 26, 2 × 26, 3 × 26, 4 × 26, was used to reconstruct convex and concave phantoms based on simulated detector measurements, as shown in Figure 7 and Figure 8 for the normalized values, respectively.

A set of planar symmetric Gaussian functions with a uniform distribution in the approximation region was employed for the reconstruction of the plasma emissivity. The parameters of the Gaussian base functions, namely *position* and *sigma*, were selected automatically for a given set in accordance with the requisite criteria.

**Figure 9:**
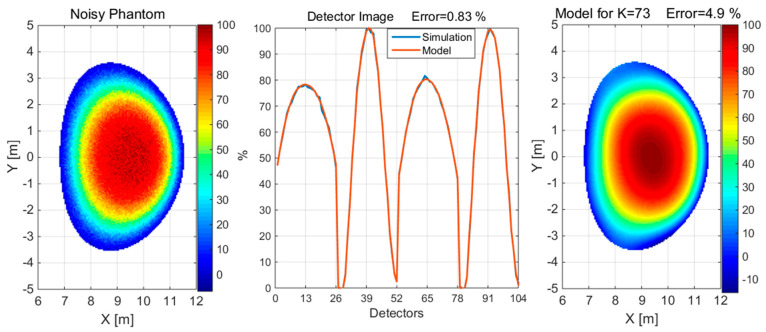
The reconstruction of the convex phantom with 5% noise when measured with 1% noise using a detection set consisting of 4 × 26 detectors. The value of K is the number of Gaussian basis functions used for the approximation.

**Figure 10:**
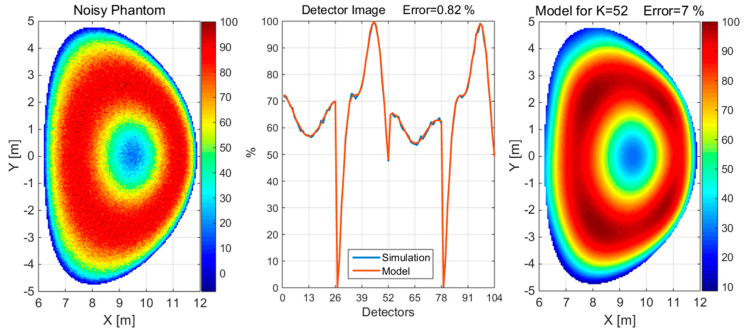
Reconstruction of a concave with 5% noise when measured with 1% noise using a detection set consisting of 4 × 26 detectors. The value of K is the number of Gaussian basis functions used for the approximation.

In the next steps of the reconstruction validation, both convex and concave phantoms were distorted by 5% noise; in addition, the simulated detector measurement was distorted by 1% noise. The reconstruction and the corresponding detector images, generated using a detection set of 4 × 26 detectors, are shown in Figure 9 and Figure 10, respectively, for normalized values.

The effectiveness of the reconstruction of the tomographic plasma image depends on the configuration of the detection system. For a specific range of parameters of the basis function, the phantom reconstruction gives a stable solution with moderate relative error, while the matching of the measurement simulation to its model for the detector is quite precise.

## 5. First Approximation: Implementation of Constraints Based on Magnetic Field Configuration

The deployment of more extensive arrays of detectors from different directions and the imposition of additional constraints may be required in order to accommodate more complex and realistic emissivity distributions. In the reconstruction of the emissivity of plasma in a tokamak, the dependencies related to the magnetic field configuration are frequently taken into account. It is assumed that the plasma properties remain constant on magnetic flux surfaces and can be represented as one-dimensional profiles that depend on poloidal magnetic flux Ψ. The reconstruction was based on the assumption that the distribution of radiation intensity can be described as a specific function of magnetic flux V(Ψ). This condition is of great importance in tomography, effectively reducing the problem of reconstruction to a one-dimensional problem for a known magnetic field configuration. Ultimately, for a specified planar magnetic field distribution Ψ(P), the emissivity distribution relationship can be derived as a complex function $V\left[\Psi \left(P\right)\right]=W\left(P\right)$ across the entire plasma cross-section in the domain of all points P. Based on the data obtained from the detectors, relationship V(Ψ) should be determined. In order to reconstruct function V(Ψ), a linear profile model v(Ψ) is assumed. This is described by a set of base functions {$F_{k}\left(\Psi \right)$} for a set of K coefficients {$a_{k}$}:

(6) $$V\left(\Psi \right)≅v\left(\Psi \right)=\sum_{k=1}^{K}a_{k}F_{k}\left(\Psi \right).$$

It can be demonstrated that for complex functions $v\left[\Psi \left(P\right)\right]$ = $w\left(P\right)$ and $F_{k}\left[\Psi \left(P\right)\right]=E_{k}\left(P\right)$, an analogous relationship is obtained using Equation (2).

A discrete model defined by vectors $V\left(\Psi \right)\left[L\times 1\right]$ and $v\left(\Psi \right)\left[L\times 1\right]\;$for selected L field values Ψ can be represented by a set of base functions $F\left(\Psi \right)\left[L\times K\right]$ for K vector components $F_{k}\left(\Psi \right)$. Similarly, a discrete model defined by vectors $w\left(P\right)\left[M\times 1\right]$ and $W\left(P\right)\left[M\times 1\right]$ for M points P of the measurement grid can be represented by a set of base functions as a matrix $E\left(P\right)\left[M\times K\right]\;$for K component vectors **E**_k_(P).

The set of unknown coefficients {$a_{k}$} can be expressed as a vector $A\left[K\times 1\right]$. This results in a reduction of the original number of unknown values for the magnetic field lines, as L is reduced to number K of unknown coefficients for the given base functions. Finally, the equations are represented in matrix form:

(7) $$V(\Psi )≅v\left(\Psi \right)=F\left(\Psi \right)\cdot A\mathrm{and}\;W(P)≅w\left(P\right)=E\left(P\right)\cdot A.$$

The emissivity distribution **W** and the **w** model are transformed in the detection system using Equation (4). The model mapping sequence v→w→z corresponds to a series of transformations of the K base functions, as outlined in the following scheme:

(8) $$F\left[L\times K\right]\overset{\Psi (P)}{\Rightarrow }E\left[M\times K\right]\overset{H}{\Rightarrow }G[N\times K]$$

The tomographic task is reduced to the vector estimation of unknown parameter **A** based on the measurement data of the **Z** detector in accordance with Formula (5) and the application of Equation (3) to reconstruct the emissivity distribution.

## 6. Results of the Validation of the Reconstruction Method in the Context of Magnetic Field Constraints

**Figure 11:**
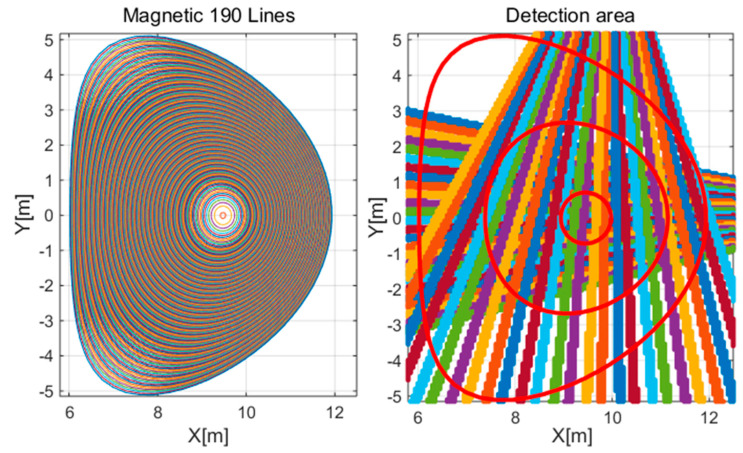
Distribution of magnetic field surfaces and the detection arrangement for two sets of 26 × 26 detectors. The colored and red closed lines in the figures depict the magnetic field surfaces.

A consequence of the assumption of the magnetic dependence of plasma emissivity is the existence of specific areas with constant emissivity values in the vicinity of magnetic lines. In conclusion, the radiation intensity and the model described by the base functions are dependent on the numerical index of the corresponding magnetic field surface (presented as lines in plasma cross section). The configuration of magnetic field surfaces (ITER baseline scenario) [6] and the detection system comprising two sets of 26 × 26 detectors for a measurement grid of 134 × 206 with a resolution of 5 cm are presented in Figure 11. To enhance the precision of the reconstruction based on the aforementioned distribution of magnetic field lines, they can be condensed through interpolation.

**Figure 12:**
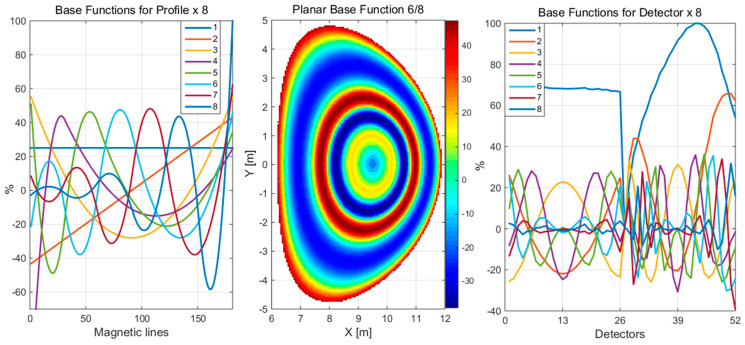
The polynomial spline utilizing eight base functions, as outlined in the scheme of Formula (8).

A number of options are available for the selection of effective basis functions for the approximation of one-dimensional profiles. A series of tests were conducted to evaluate the efficacy of an approximation method based on a set of Gaussian functions with *position* and *sigma* parameters and spline polynomials for the reconstruction of two reference plasma phantoms in a system of 26 × 26 detectors. The methodology for generating spline polynomials with parameterized nodes and degree is outlined in [22]. The polynomial spline base functions, as outlined in the scheme of Formula (8), are presented in Figure 12 as examples. The solution obtained for various types of base functions within a specified range of parameters provides stable and comparable results. The parameters of the base functions were selected appropriately in the automatic review process for a given set.

**Figure 13:**
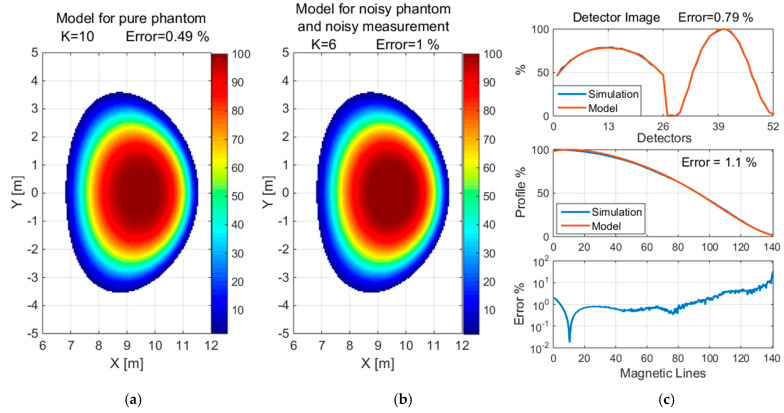
Reconstruction of the convex phantom: (**a**) without noise (**b**) with 5% noise when measured with 1% noise, (**c**) detector image, profile graph and error depending on magnetic lines. The value of K is the number of basis functions used for the approximation.

**Figure 14:**
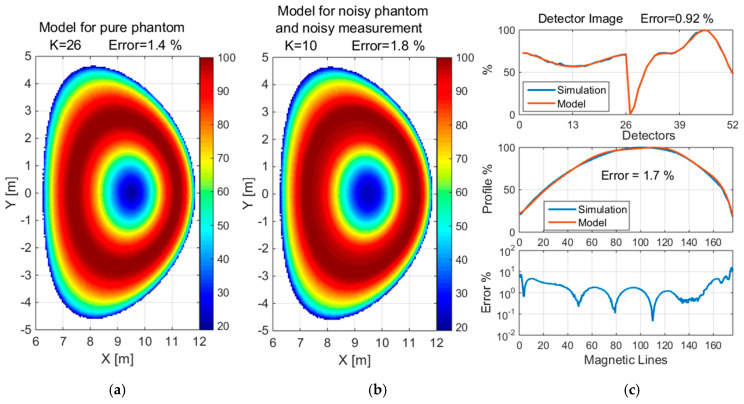
Reconstruction of the concave phantom: (**a**) without noise, (**b**) with 5% noise when measured with 1% noise, (**c**) detector image, profile graph and error depending on magnetic lines. The value of K is the number of basis functions used for the approximation.

The reconstruction of the convex and concave phantom without noise and with 5% noise when measured with 1% noise is shown in Figure 13 and Figure 14, respectively, for normalized values. Additionally, the detector images for simulated and reconstructed measurements and the profile graph and error depending on the magnetic lines are presented.

## 7. Summary

The proposed method of reconstructing the radiation distribution is based on a linear planar distribution model, which is described by a set of base functions. Tests based on planar base functions demonstrated a significant dependence of the solution on the detector arrangement and the parameters of the base functions. Tests for relatively simple image shapes yielded moderately positive results. However, more complex shapes required additional assumptions and constraints. Tests with perturbations from additional noise images demonstrated the smoothing capabilities of the basis function model and effective estimation of the original data. Nevertheless, in the case of lower approximation accuracy and higher levels of noise, subsequent reconstruction realizations may vary, which may require averaging.

In this study, the initial approximation to reconstruct the plasma emissivity in a tokamak was to consider that plasma radiation is assigned to the magnetic field surfaces. It was assumed that plasma emissivity would be constant on magnetic flux surfaces and could be represented as one-dimensional profiles depending on the poloidal magnetic flux. In reconstructing the plasma emissivity distribution, three sets of base functions were considered: spline polynomials and Gaussian functions with *sigma* and *position* parameters. The reconstruction error of the two simulated reference phantoms was found to be a function of the number of detectors and was found to be in range of 1–2%, which suggests that reliable identification of the actual plasma intensity distributions in the tokamak is possible. The results obtained by this method are comparable to those produced by other techniques, although the calculation procedure appears to be less complex and therefore more suitable for real-time measurements needed in future fusion reactors. Further studies are planned to incorporate additional considerations and developments, including more realistic assumptions about the dependence of plasma radiation distribution on other plasma parameters. For instance, more complex radiation dependence on factors such as magnetic flux surfaces will be taken into account.

## Data Availability

Data are contained within the article.
